# Mesenteric Mixed Type of Castleman Disease: A Report of a Rare Case

**DOI:** 10.7759/cureus.61735

**Published:** 2024-06-05

**Authors:** Mohamed Chablou, Assia Elberhoumi, Abdellah Haddadi, Anass Idrissi, Mohamed Kafih

**Affiliations:** 1 General Surgery, Provincial Hospital Center, Fquih Ben Salah, MAR; 2 Radiology, Private Hospital Oliviers-Yasmine, Fquih Ben Salah, MAR; 3 General Surgery, Private Hospital Oliviers-Yasmine, Fquih Ben Salah, MAR; 4 Visceral Surgery, Faculty of Medicine, Mohammed VI University of Health Sciences, Casablanca, MAR

**Keywords:** multicentric, unicentric, surgery, mesenteric mass, castleman's disease

## Abstract

Castleman disease is a rare type of lymph node hyperplasia primarily affecting the mediastinum, with mesenteric localization being extremely uncommon. It is classified into solitary and multicentric forms. In this case report, we present the case of a 46-year-old female patient in whom an incidental mesenteric mass was discovered during the workup for a ventral hernia. The mass was completely excised, and the histopathological examination confirmed the diagnosis of mixed-type Castleman disease. Surgery is the treatment of choice for localized forms of this condition, and histological examination is crucial in confirming the diagnosis.

## Introduction

Castleman disease is a rare entity, a lymphoproliferative disorder characterized by hyperplasia of the lymph nodes [1]. Its etiology remains unknown. The most frequently affected site is the mediastinum, while mesenteric localization is rare. Clinically, it is classified into a solitary (localized) form, often discovered incidentally and with a good prognosis, and a multicentric form with a guarded prognosis. A newly described subgroup, called oligocentric, presents with a clinical evolution similar to the multicentric form [2]. The preoperative diagnosis of mesenteric Castleman disease can be difficult due to the nonspecific clinical and radiological presentation, and histological confirmation remains essential. Surgery plays a predominant role in the management of this pathology. We report the rare case of a 46-year-old woman admitted to our hospital for management of a ventral hernia, where radiological examinations revealed a mesenteric mass. The diagnosis of mixed-type Castleman disease was made postoperatively after histopathological examination.

## Case presentation

**Figure 1:**
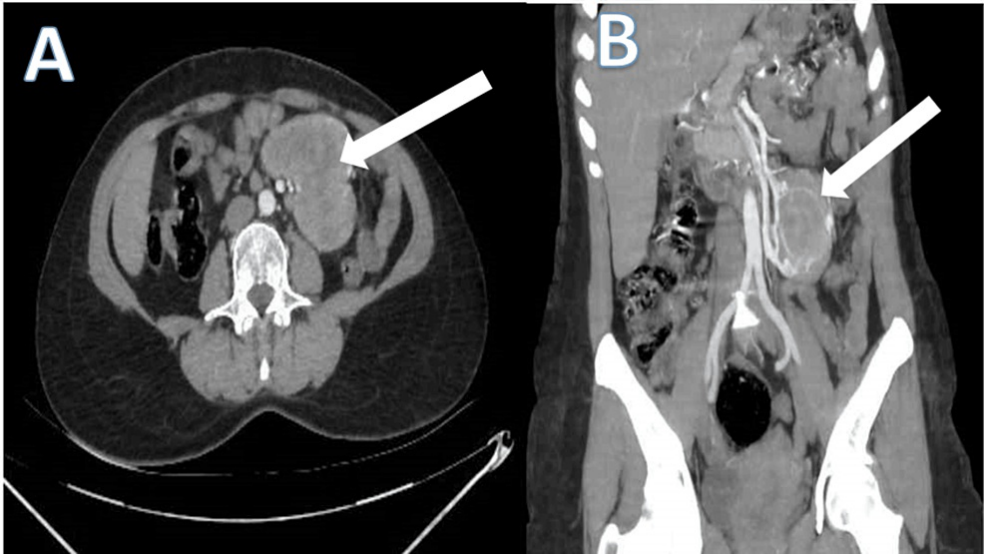
Abdominal CT showing a mesenteric mass with intense and heterogeneous contrast enhancement (white arrow) (A) Sectional axial image. (B) Coronal section.

A 46-year-old female with no significant past medical history presented to our hospital for the management of an uncomplicated ventral hernia. Clinical examination revealed a reducible supraumbilical ventral hernia with no palpable mass or lymphadenopathy. An abdominal CT scan, performed as part of the hernia workup, showed a 14-mm supraumbilical fascial defect as well as a hypodense, intensely and heterogeneously enhancing mesenteric mass (Figure 1).

**Figure 2:**
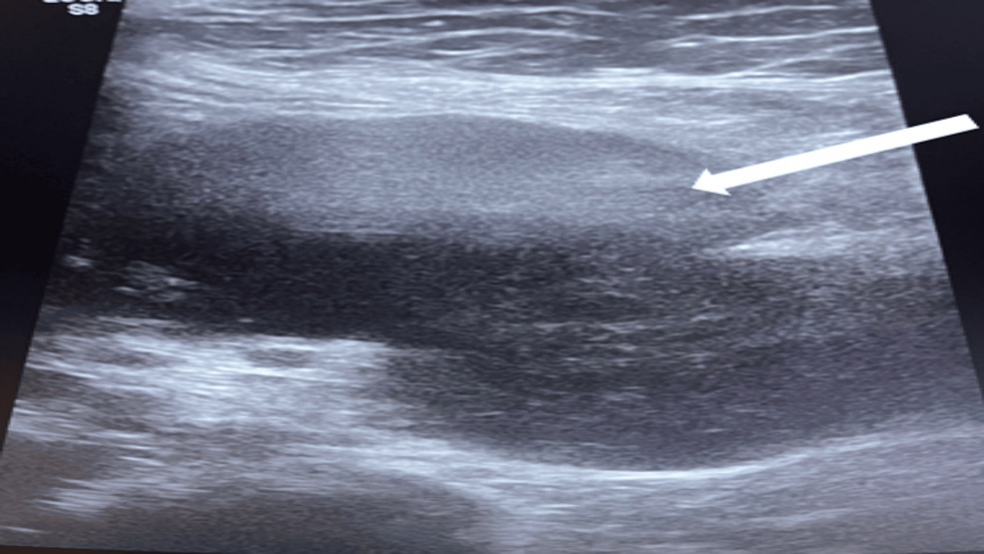
Ultrasound image showing a bilobed, hypoechoic, heterogeneous mesenteric mass (white arrow)

Complementary abdominal ultrasound demonstrated a retro-umbilical, bilobed, hypoechoic, heterogeneous intra-peritoneal mass measuring 64 × 52 mm (Figure 2).

**Figure 3:**
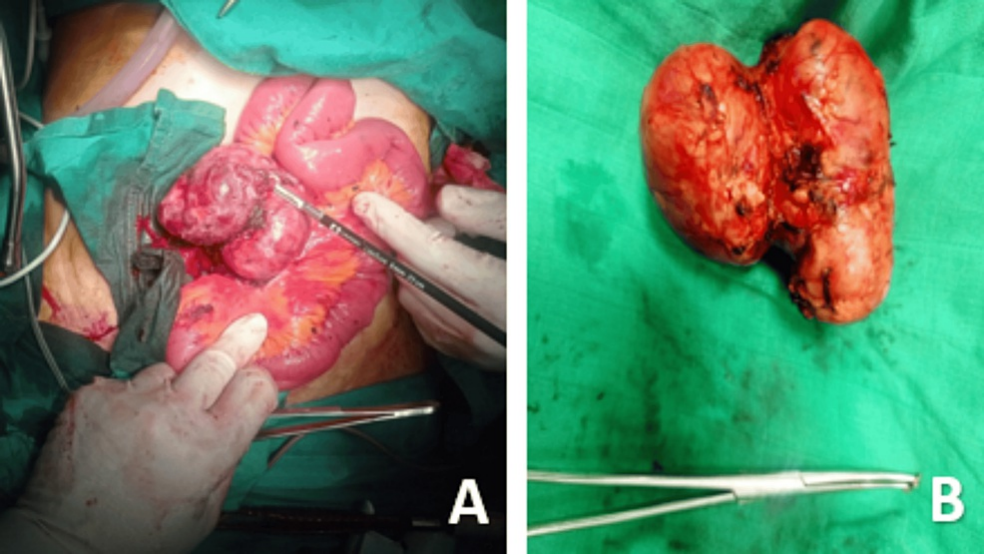
Surgical images showing the complete resection of the mesenteric mass (A) Intraoperative image. (B) Surgical specimen.

Routine laboratory investigations were unremarkable. The cervicothoracic CT scan did not reveal any additional sites of disease involvement. The patient underwent laparoscopic surgery, converted to laparotomy, during which the mesenteric mass was completely resected, along with superior mesenteric lymph node dissection and hernia repair (Figure 3).

Histopathological examination of the surgical specimen revealed mixed-type Castleman disease with plasmacytic and fibro-hyaline components. The patient was discharged two days later. HIV and human herpesvirus (HHV-8) serologies were requested and found to be negative. Postoperative follow-up consultations were conducted at three-month intervals over the course of one year for the patient, during which no recurrence of mesenteric pathology was observed.

## Discussion

Castleman disease is a rare disorder, first described in 1956 [3]. Other terminology used for this condition includes angiofollicular lymph node hyperplasia or giant lymph node hyperplasia [4]. The mediastinum remains the most common site, with the disease frequently developing along the tracheobronchial and hilar lymph node chains. However, other sites can be involved [5-7]. Abdominal and pelvic localizations are uncommon, occurring in 5.7% of cases (3.5% in the mesentery) and 6.6% retroperitoneally [8]. There is no gender predilection.

Although infrequent, the pathogenesis of Castleman disease remains complex. Several hypotheses suggest it may be due to a hyperplastic reactive response to an antigenic stimulus (virus: HHV-8 or immunodeficiency associated with HIV) [9] or a hamartomatous process. Others propose a chronic inflammatory reaction due to increased IL-6 production [10-12].

Unicentric Castleman disease is limited to a single site, often asymptomatic and an incidental finding [3-5], as in the present case. The multicentric form involves multiple lymph node regions and is a progressive disease that can be life-threatening [13]. This form is sometimes associated with HIV infection [14] and Kaposi’s sarcoma [3]. The oligocentric form involves more than two adjacent lymph node stations and exhibits a clinical course similar to the multicentric form [2].

This rare disease poses a diagnostic challenge, as it is often asymptomatic and an incidental finding, as in our patient. Other clinical signs, such as fever, fatigue, night sweats, and abdominal pain, are nonspecific and can mimic neoplasms.

Morphological examinations alone are insufficient to establish the definitive diagnosis, as mesenteric localization is rare and radiological findings are not pathognomonic or specific, potentially leading to confusion with other pathologies such as gastrointestinal stromal tumors, lymphomas, leiomyomas, and leiomyosarcomas. In our patient, abdominal CT revealed a hypodense, intensely and heterogeneously enhancing mesenteric mass, while complementary abdominal ultrasound showed a retro-umbilical, bilobed, hypoechoic, heterogeneous intraperitoneal mass measuring 64 × 52 mm. Other imaging modalities, like MRI [15], may be requested based on the disease location and diagnostic orientation.

Given this clinical and radiological heterogeneity, histological proof is necessary to confirm the diagnosis. Histologically, the hyaline vascular form is most common, followed by the plasmacytic (10%) and mixed forms, the latter being very rare and the type diagnosed in our patient [5].

Surgery remains the treatment of choice for Castleman disease, as complete resection can be curative, particularly for localized disease. It is also necessary for diagnostic purposes; in our patient, the definitive diagnosis was established postoperatively after histopathological examination. Other therapeutic alternatives, such as radiation therapy and chemotherapy, may be useful but are not curative [16,17]. After confirming the diagnosis of Castleman disease in our patient, HIV and HHV8 serologies were requested and found to be negative.

Although rare, Castleman disease should be included in the differential diagnoses for mesenteric masses.

## Conclusions

Mesenteric localization of Castleman disease is exceedingly rare, and its clinical and radiological presentation does not allow for a definitive diagnosis. A complete surgical excision represents the optimal treatment, and histological examination confirms the diagnosis. Castleman disease should be included in the differential diagnoses for mesenteric masses.
